# CXCR7 is induced by hypoxia and mediates glioma cell migration towards SDF-1α

**DOI:** 10.1186/1471-2407-13-347

**Published:** 2013-07-17

**Authors:** Mine Esencay, Yasmeen Sarfraz, David Zagzag

**Affiliations:** 1 Microvascular and Molecular Neuro-oncology Laboratory, New York University Langone Medical Center, New York, NY, USA; 2 Department of Pathology, New York University Langone Medical Center, New York, NY, USA; 3 Division of Neuropathology, New York University Langone Medical Center, New York, NY, USA; 4 Department of Neurosurgery, New York University Langone Medical Center, New York, NY, USA; 5 New York University School of Medicine, New York University Langone Medical Center, 550 First Avenue, New York, NY 10016, USA

**Keywords:** Glioma, Hypoxia, CXCR4, CXCR7, Migration

## Abstract

**Background:**

Glioblastomas, the most common and malignant brain tumors of the central nervous system, exhibit high invasive capacity, which hinders effective therapy. Therefore, intense efforts aimed at improved therapeutics are ongoing to delineate the molecular mechanisms governing glioma cell migration and invasion.

**Methods:**

In order to perform the studies, we employed optimal cell culture methods and hypoxic conditions, lentivirus-mediated knockdown of protein expression, Western Blot analysis, migration assays and immunoprecipitation. We determined statistical significance by unpaired t-test.

**Results:**

In this report, we show that U87MG, LN229 and LN308 glioma cells express CXCR7 and that exposure to hypoxia upregulates CXCR7 protein expression in these cell lines. CXCR7-expressing U87MG, LN229 and LN308 glioma cells migrated towards stromal-derived factor (SDF)-1α/CXCL12 in hypoxic conditions in the Boyden chamber assays. While shRNA-mediated knockdown of CXCR7 expression did not affect the migration of any of the three cell lines in normoxic conditions, we observed a reduction in the migration of LN229 and LN308, but not U87MG, glioma cells towards SDF-1α in hypoxic conditions. In addition, knockdown of CXCR7 expression in LN229 and LN308 glioma cells decreased levels of SDF-1α-induced phosphorylation of ERK1/2 and Akt. Inhibiting CXCR4 in LN229 and LN308 glioma cells that were knocked down for CXCR7 did not further reduce migration towards SDF-1α in hypoxic conditions and did not affect the levels of phosphorylated ERK1/2 and Akt. Analysis of immunoprecipitated CXCR4 from LN229 and LN308 glioma cells revealed co-precipitated CXCR7.

**Conclusions:**

Taken together, our findings indicate that both CXCR4 and CXCR7 mediate glioma cell migration towards SDF-1α in hypoxic conditions and support the development of therapeutic agents targeting these receptors.

## Background

CXCR4 is a well-known G-protein coupled receptor (GPCR) for the small chemokine stromal-derived factor (SDF)-1α, which is also known as CXCL12. Another GPCR, CXCR7, has been identified as a second receptor for SDF-1α. This receptor was originally cloned based on its homology with conserved domains of GPCRs and named as “RDC1” [1]. At the beginning, it was believed to be a receptor for vasointestinal peptide, but later reports dismissed this possibility [2]. Combined phylogenetic and chromosomal location studies revealed the structural resemblance of the orphan receptor RDC1 to CXC chemokine receptors and implicated CXC chemokines as potential ligands [1]. It was shown that RDC1 could serve as a co-receptor for human immunodeficiency virus and simian immunodeficiency virus strains, just like CXCR4 [3]. Soon afterwards, SDF-1α was shown to bind with high affinity to and signal through the orphan receptor RDC1 [2], leading to the designation of the receptor as “CXCR7”.

CXCR7 is expressed on vascular endothelial cells, T cells, dendritic cells, B cells, brain-derived cells and tumor cells, including human glioma cells [2-4]. Its expression is upregulated by hypoxia in human microvascular endothelial cells [5]. CXCR7 plays an important role in several carcinomas, including breast cancer, lung cancer, and prostate cancer [6,7]. Immunohistochemical staining of metastatic melanoma sections demonstrated CXCR7 staining on tumor cells [5]. This receptor is believed to play a pivotal role in growth, adhesion, survival, angiogenesis, and invasion of tumor cells [2,6,7]. Administration of a small molecule antagonist of CXCR7 correlated with reduced tumor size in both xenograft and syngeneic *in vivo* tumor growth studies [6]. Ectopic expression of the receptor has been shown to enhance tumor formation in nude mice *in vivo*[8]. A recent study demonstrated that in prostate cancer, CXCR7 potentially promotes invasion through its downstream targets of CD44 and cadherin-11 [7]. Balabanian and colleagues showed that SDF-1α-induced T cell migration was dependent on both CXCR4 and CXCR7, and combined inhibition of these two receptors resulted in additive inhibitory effects on the migration of T cells [2].

Hypoxia is a major player in the microenvironment of gliomas that orchestrates adaptive responses by stimulating the expression of several genes involved in tumorigenesis. However, despite accumulating data, the regulation of CXCR7 by hypoxia and its contribution to glioma migration have not been fully elucidated yet. Here, we show that U87MG, LN229 and LN308 glioma cells express CXCR7 and exposure to hypoxia upregulates CXCR7 protein expression in these cell lines. CXCR7-expressing U87MG, LN229 and LN308 glioma cells migrated towards SDF-1α in hypoxic conditions in the Boyden chamber assays. While shRNA-mediated knockdown of CXCR7 expression did not affect the migration of any of the three cell lines in normoxic conditions, we observed a reduction in the migration of LN229 and LN308, but not U87MG, glioma cells towards SDF-1α in hypoxic conditions. In addition, knockdown of CXCR7 expression in LN229 and LN308 glioma cells decreased levels of SDF-1α-induced phosphorylation of ERK1/2 and Akt. Inhibiting CXCR4 in LN229 and LN308 glioma cells that were knocked down for CXCR7 did not further reduce migration towards SDF-1α in hypoxic conditions and did not affect the levels of phosphorylated ERK1/2 and Akt. Analysis of immunoprecipitated CXCR4 from LN229 and LN308 glioma cells revealed co-precipitated CXCR7. Taken together, our findings indicate that both CXCR4 and CXCR7 mediate glioma cell migration towards SDF-1α in hypoxic conditions.

## Results

### Hypoxia upregulates CXCR7 protein expression

**Figure 1:**
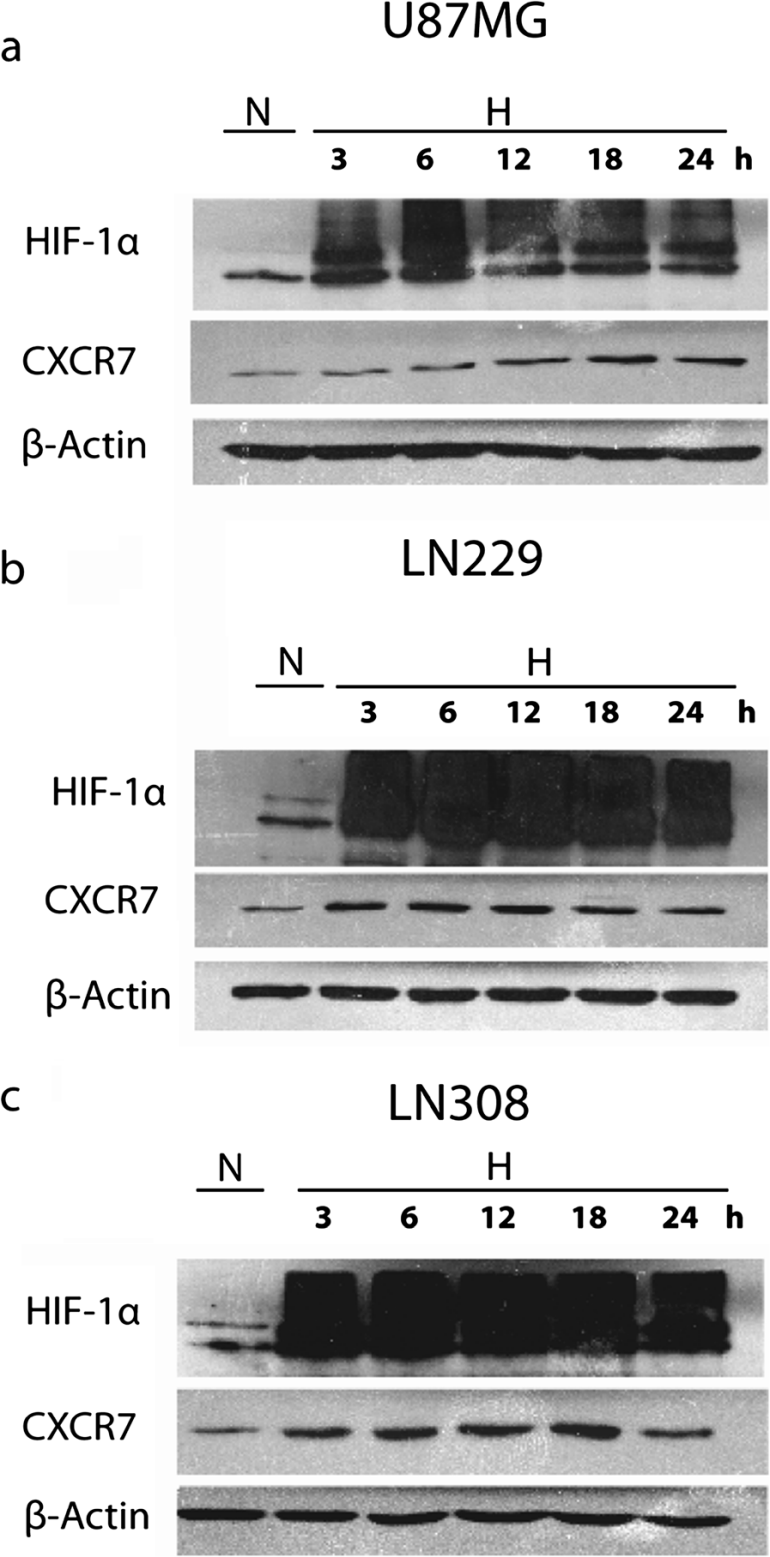
**Hypoxia upregulates CXCR7 protein expression*. *(a)** U87MG, **(b)** LN229 and **(c)** LN308 glioma cells were cultured in normoxic or hypoxic conditions for 3, 6, 12, 18 and 24 h. Total cell lysates were collected and analyzed by Western blot for HIF-1α and CXCR7 protein expression. β-Actin was used as loading control. Data are representative of two independent experiments with similar results. N, normoxia (20% O_2_); H, hypoxia (1% O_2_).

We first determined the effect of hypoxia on CXCR7 protein expression in glioma cells. U87MG, LN229 and LN308 glioma cells were cultured in normoxic or hypoxic conditions for 3, 6, 12, 18 and 24 h. Total cell lysates were collected and subjected to Western blot analysis (Figure 1). We observed that U87MG, LN229 and LN308 glioma cells expressed CXCR7. Exposure to hypoxia increased HIF-1α and CXCR7 protein levels in all cell lines. In LN229 (Figure 1b) and LN308 (Figure 1c) glioma cells, hypoxia upregulated CXCR7 protein expression immediately, starting at 3 h and declining after 18 h. Conversely, in U87MG (Figure 1a) glioma cells, hypoxia upregulated CXCR7 protein expression at 18 h, declining slowly thereafter. CXCR7 protein expression was upregulated significantly by two-fold in U87MG and LN229, and three-fold in LN308 glioma cells at 18 h.

### CXCR7 mediates the migration of LN229 and LN308 glioma cells towards SDF-1α in hypoxic conditions

We have previously shown that CXCR4-positive glioma cells increase their migration towards SDF-1α [9]. Both CXCR4 and CXCR7 are receptors for SDF-1α. Therefore, we wished to evaluate the role of CXCR7 in glioma cell migration towards SDF-1α in normoxic and hypoxic conditions. For this purpose, we first knocked down the expression of CXCR7 in U87MG, LN229 and LN308 glioma cells using a lentivirus-mediated shRNA vector directed against the receptor. As control, cells were infected with a lentivirus-mediated shRNA vector directed against LacZ. The efficiency of knockdown was confirmed by Western blot analysis (data not shown). We selected two sequences that effectively knocked down the expression of the receptor, S4 and S5, and tested them both in the following migration experiments to ensure consistent results.

**Figure 2:**
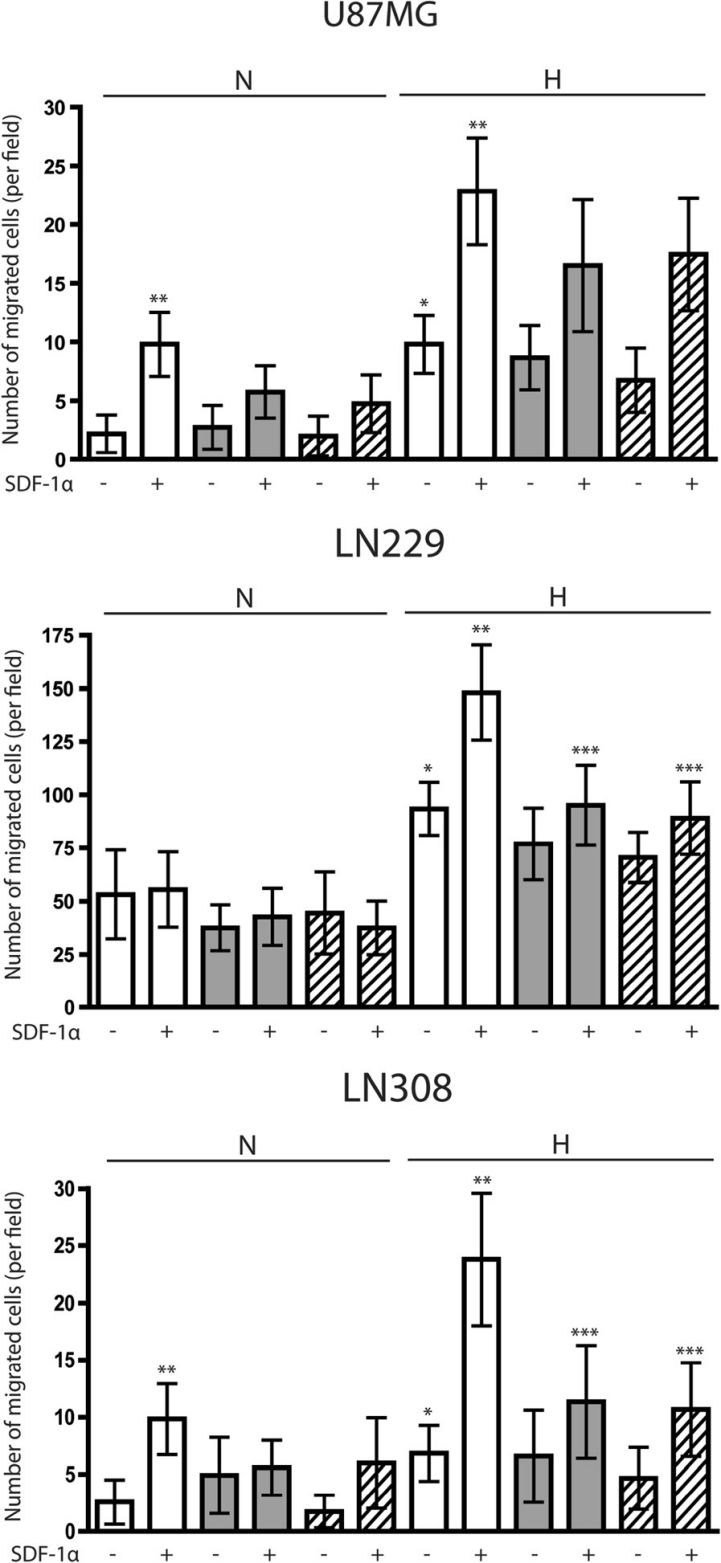
**CXCR7 mediates the migration of LN229 and LN308 glioma cells towards SDF-1α in hypoxic conditions.** shRNA-infected U87MG, LN229 and LN308 glioma cells were seeded in migration chambers in the presence or absence of 100 ng/ml (10 nM) of SDF-1α in the lower well. They were allowed to migrate for 8 h in normoxic or hypoxic conditions. Bar graphs indicate the average number of migrated cells per field. Error bars denote mean ± standard deviation. ^*^P<0.001 versus normoxic control; ^**^P<0.001 versus non-SDF-1α exposed cells; ^***^P<0.001 versus SDF-1α exposed hypoxic cells. Bar graphs represent pooled data from two independent experiments. N, normoxia (20% O_2_); H, hypoxia (1% O_2_); white bars, shLacZ; grey bars, shCXCR7 S4; hatched bars, shCXCR7 S5.

To test whether CXCR7 knockdown reduces the number of migrated cells towards SDF-1α, shRNA-infected U87MG, LN229 and LN308 glioma cells were seeded in migration chambers in the presence or absence of 100 ng/ml of SDF-1α in the lower well. They were allowed to migrate for 8 h in normoxic or hypoxic conditions. After fixing and staining, the number of migrated cells was quantitated. Results from two independent experiments are shown (Figure 2). First, we observed that in hypoxic conditions, all cell lines increased their migration significantly compared to similar cultures in normoxic conditions (P< 0.001). Both in normoxic and hypoxic conditions, and in the presence of SDF-1α in the lower well, U87MG and LN308 glioma cells showed a significant increase in migration towards SDF-1α compared to control cultures (P< 0.001). By contrast, LN229 glioma cells increased their migration towards SDF-1α only in hypoxic conditions (P<0.001). In normoxic conditions, knockdown of CXCR7 expression did not inhibit the increased migration of glioma cells towards SDF-1α. However, in hypoxic conditions, knockdown of CXCR7 expression significantly reduced the number of migrated LN229 and LN308, but not U87MG, glioma cells towards SDF-1α as compared to control cultures (P<0.001). This is consistent with our observation that CXCR7 is not significantly induced by hypoxia in U87MG cells during the 8 h incubation period. Hypoxia upregulates CXCR7 in U87MG glioma cells at 18 h (Figure 1).

### Inhibiting CXCR4 in glioma cells that are knocked down for CXCR7 does not further reduce migration towards SDF-1α

**Figure 3:**
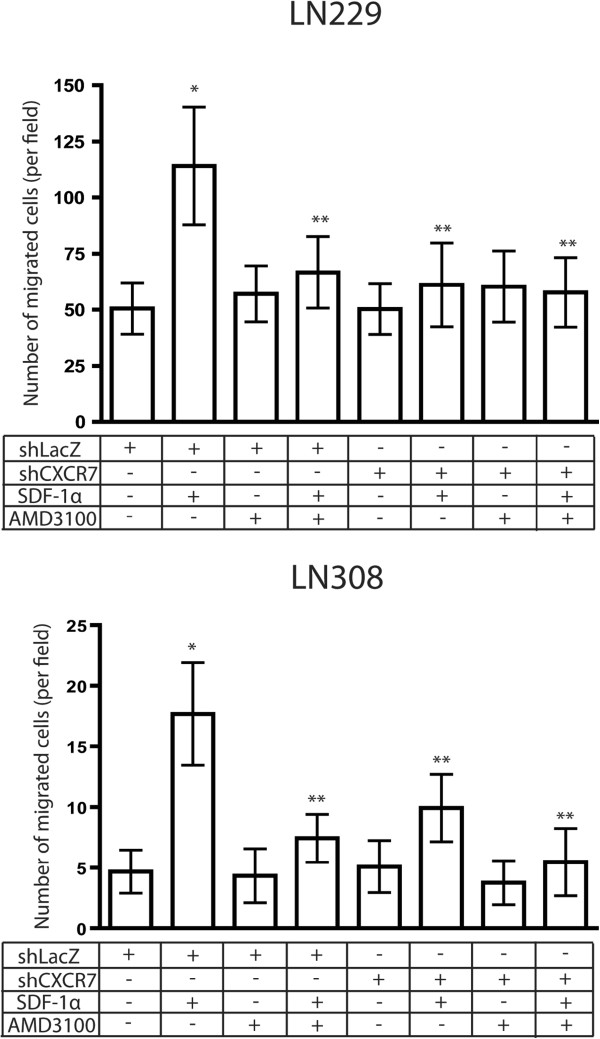
**Inhibiting CXCR4 in glioma cells that are knocked down for CXCR7 does not further reduce migration towards SDF-1α.** shRNA-infected LN229 and LN308 glioma cells were seeded in migration chambers with or without 100 nM of AMD3100 and in the presence or absence of 100 ng/ml (10 nM) of SDF-1α in the lower well. They were allowed to migrate for 8 h in hypoxic conditions (1% O_2_). Bar graphs indicate the average number of migrated cells per field. Error bars denote mean ± standard deviation. ^*^P<0.001 versus non-SDF-1α exposed cells; ^**^P<0.001 versus SDF-1α exposed cells. Bar graphs represent pooled data from two independent experiments.

We have previously shown that AMD3100, a CXCR4 inhibitor, decreases glioma cell migration towards SDF-1α [9]. Since we observed that knockdown of CXCR7 expression similarly decreased migration towards SDF-1α, we tested whether combined inhibition of these two receptors resulted in further reduction in the number of migrated glioma cells towards SDF-1α. According to previous results, knockdown of CXCR7 expression reduced the migration of only LN229 and LN308 glioma cells towards SDF-1α at 8 h of incubation period, and only in hypoxic conditions. Therefore, we carried out the rest of migration studies according to these results. shRNA-infected LN229 and LN308 glioma cells were seeded in migration chambers with or without 100 nM of AMD3100 and in the presence or absence of 100 ng/ml of SDF-1α in the lower well. They were allowed to migrate for 8 h in hypoxic conditions. After fixing and staining, the number of migrated cells was quantitated. Results from two independent experiments are shown (Figure 3). Consistent with our earlier observations, migration of both LN229 and LN308 glioma cells increased significantly towards SDF-1α as compared to control cultures (P<0.001). Both AMD3100 and knockdown of CXCR7 expression significantly inhibited the increased migration of glioma cells towards SDF-1α (P<0.001). However, inhibiting CXCR4 in LN229 and LN308 glioma cells that were knocked down for CXCR7 expression did not further reduce migration towards SDF-1α.

### SDF-1α induces CXCR7-mediated phosphorylation of ERK1/2 and Akt in LN229 and LN308 glioma cells

**Figure 4:**
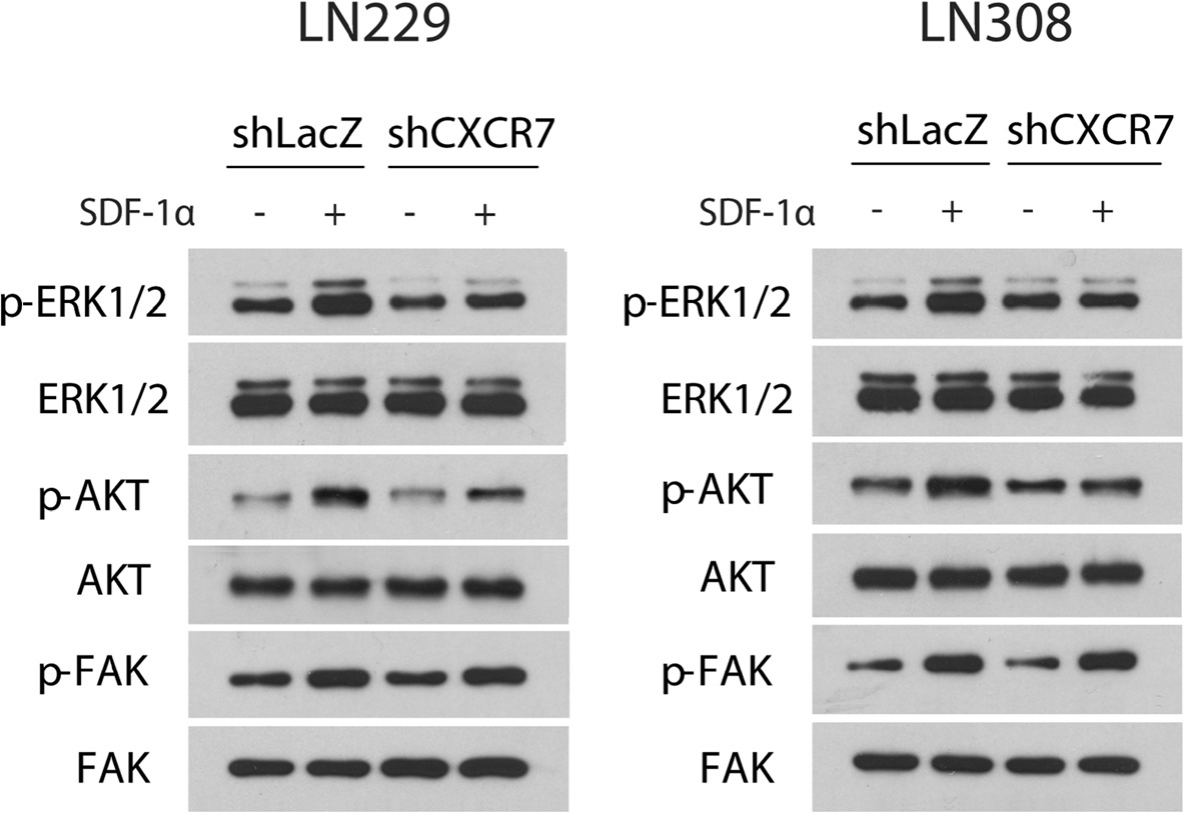
**SDF-1α induces CXCR7-mediated phosphorylation of ERK1/2 and Akt in LN229 and LN308 glioma cells.** shRNA-infected LN229 and LN308 glioma cells were exposed to SDF-1α for 15 min and analyzed for total and phosphorylated ERK1/2, Akt and FAK by Western blot analysis. Data represent one of two independent experiments.

As we mentioned above, phosphorylated ERK1/2, Akt and FAK play critical roles in glioma cell migration and invasion. We previously provided evidence that SDF-1α induces phosphorylation of ERK1/2, Akt and FAK in LN308 glioma cells that display CXCR4-mediated migration towards SDF-1α [9]. As a first step to elucidate molecular signaling pathways mediated by CXCR7, we tested whether SDF-1α induces phosphorylation of ERK1/2, Akt and FAK in LN229 and LN308 glioma cells that demonstrate CXCR7-mediated migration towards SDF-1α. LN229 and LN308 glioma cells infected with shRNA vector directed against CXCR7 or LacZ were exposed to SDF-1α for 15 min and analyzed for total and phosphorylated ERK1/2, Akt and FAK by Western blot analysis (Figure 4). We observed that SDF-1α increased the levels of phosphorylated ERK1/2, Akt and FAK two-fold, three-fold, and two-fold in LN229 and two-fold, two-fold, and three-fold in LN308 glioma cells, respectively. Knockdown of CXCR7 expression decreased the levels of SDF-1α-induced phosphorylation of ERK1/2 and Akt, but not FAK, two-fold in both glioma cell lines.

### Inhibiting CXCR4 in glioma cells that are knocked down for CXCR7 does not further reduce levels of SDF-1α-induced phosphorylation of ERK1/2 and Akt

**Figure 5:**
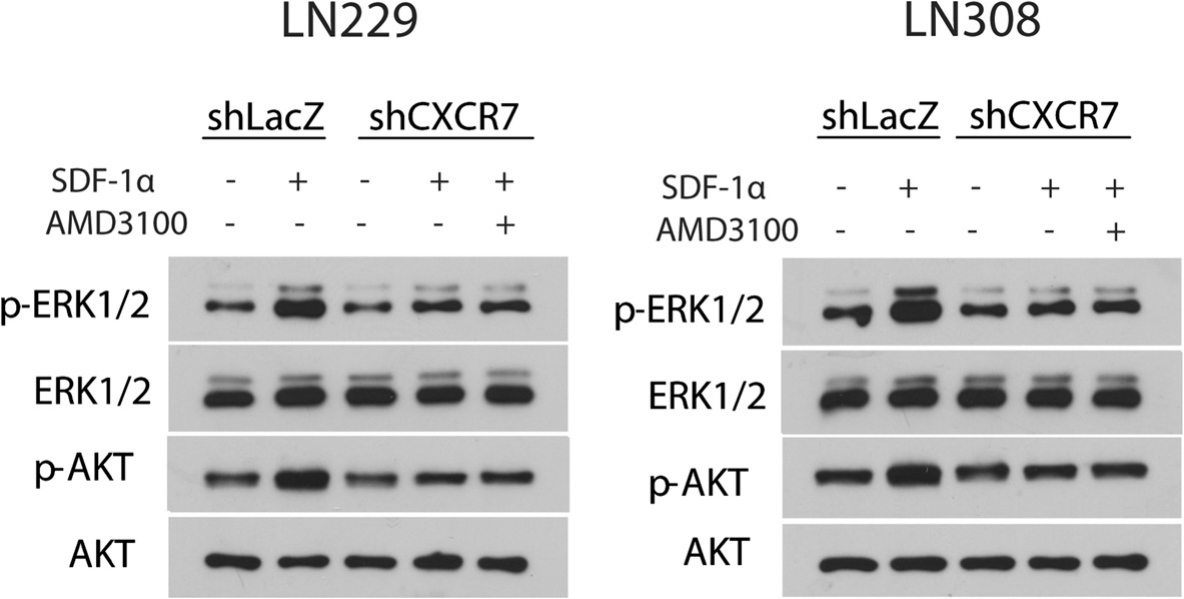
**Inhibiting CXCR4 in glioma cells that are knocked down for CXCR7 does not further reduce levels of SDF-1α-induced phosphorylation of ERK1/2 and Akt.** shRNA-infected LN229 and LN308 glioma cells were exposed to SDF-1α for 15 min in the presence or absence of 100 nM of AMD3100 and analyzed for total and phosphorylated ERK1/2 and Akt by Western blot analysis. Data represent one of two independent experiments.

Exposure of glioma cells to SDF-1α in the presence of AMD3100 decreases levels of phosphorylated ERK1/2 and Akt [9]. We thus tested whether combined inhibition of CXCR4 and CXCR7 results in further reduction in the levels of phosphorylated ERK1/2 and Akt. LN229 and LN308 glioma cells infected with shRNA vector directed against CXCR7 or LacZ were exposed to SDF-1α for 15 min in the presence or absence of 100 nM of AMD3100 and analyzed for total and phosphorylated ERK1/2 and Akt by Western blot analysis (Figure 5). Consistent with our previous observations (Figure 4), knockdown of CXCR7 expression decreased the levels of SDF-1α-induced phosphorylation of ERK1/2 and Akt two-fold in both glioma cell lines. However, inhibiting CXCR4 in LN229 and LN308 glioma cells that were knocked down for CXCR7 expression did not further reduce levels of SDF-1α-induced phosphorylation of ERK1/2 and Akt.

### CXCR4 and CXCR7 bind in glioma cells

**Figure 6:**
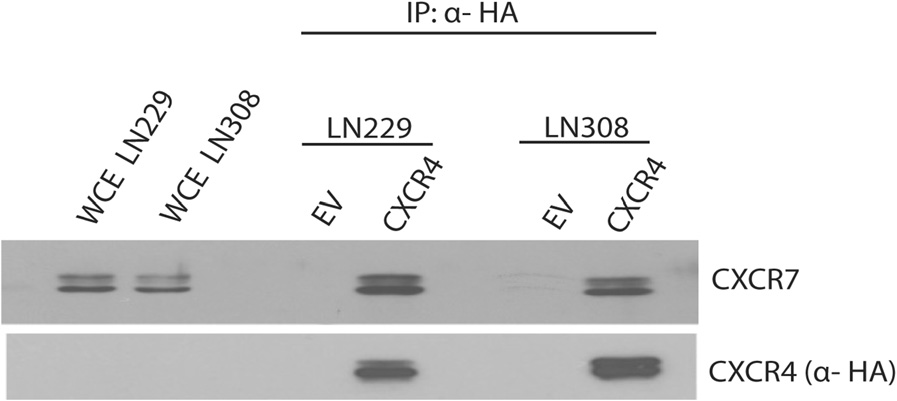
**CXCR4 and CXCR7 bind in glioma cells.** LN229 and LN308 glioma cells were transfected with an empty vector (EV) or HA-tagged CXCR4. Whole cell extracts (WCE) were immunoprecipitated (IP) with anti-HA resin and samples were subjected to Western blot analysis using anti-HA and anti-CXCR7 antibodies. Data are representative of two independent experiments with similar results.

Since our observations so far suggested a functional interaction between CXCR4 and CXCR7, we investigated the potential binding of the two receptors in glioma cells. We transfected LN229 and LN308 glioma cells with HA-tagged CXCR4 (CXCR4-HA) or an empty vector as control. We then immunoprecipitated CXCR4-HA or empty vector from LN229 and LN308 glioma cells and analyzed it for co-precipitated CXCR7 using Western blotting (Figure 6). Immunoprecipitation of CXCR4-HA led to the detection of co-precipitated CXCR7. By contrast, CXCR7 was not detectable in the empty vector.

## Discussion

Our findings demonstrate that (1) hypoxia upregulates CXCR7 protein expression in glioma cells, (2) CXCR7 mediates the migration of LN229 and LN308 glioma cells towards SDF-1α in hypoxic conditions, (3) SDF-1α induces CXCR7-mediated phosphorylation of ERK1/2 and Akt in LN229 and LN308 glioma cells, (4) inhibiting CXCR4 in glioma cells that are knocked down for CXCR7 does not further reduce either the migration towards SDF-1α or the levels of SDF-1α-induced phosphorylation of ERK1/2 and Akt, and (5) CXCR4 and CXCR7 bind in glioma cells. Collectively, our findings indicate that both CXCR4 and CXCR7 mediate glioma cell migration towards SDF-1α in hypoxic conditions.

The presence of HIF-1α binding sites beginning at −155, -1012 and −1350 base pairs upstream of the transcription initiation site of CXCR7 suggests that its expression could be regulated by hypoxia. Indeed, hypoxia-induced upregulation of CXCR7 has been reported previously in microvascular endothelial cells [5]. Our data show that the expression of CXCR7 is upregulated under hypoxic conditions in glioma cell lines. While the upregulation is evident at earlier time points of exposure to hypoxia in LN229 and LN308 glioma cells, it is not noticeable until 18 h in U87MG glioma cells. Hypoxia-mediated upregulation of CXCR7 is significant, because hypoxia is a common pathological feature of gliomas that controls the expression of many genes essential for acquisition of invasive phenotype. The invasive nature of gliomas hinders effective therapy and thus molecular mechanisms governing invasion represent attractive therapeutic targets [9]. Although many hypoxia-induced molecules that are involved in glioma biology have been elucidated, more effective design of treatment strategies warrants further identification of novel hypoxia-responsive genes that drive invasion.

Although the key role of CXCR4 in mediating SDF-1α-induced migration of glioma cells is well established [9-12], that of CXCR7, to our knowledge, has still not been confirmed. However, the discovery of CXCR7 as a second SDF-1α receptor brings to mind the possibility that CXCR7 might contribute to SDF-1α-induced migration. In a report by Balabanian et al., CXCR7 was described as a receptor that enhanced SDF-1α-dependent chemotaxis of T lymphocytes together with CXCR4 [2]. Our data support a role for CXCR7 in mediating SDF-1α-induced glioma cell migration in hypoxic conditions. Knockdown of CXCR7 expression by two independent shRNA sequences resulted in a consistent reduction in the number of LN229 and LN308, but not U87MG, glioma cells that migrated towards SDF-1α. The discrepancy observed for the U87MG cell line is attributable to the lack of hypoxia-mediated CXCR7 upregulation at 8 h of exposure to hypoxia (which is also the timeframe for the migration assays). It should also be noted that LN229 glioma cells migrated towards SDF-1α only in hypoxic conditions, where levels of CXCR4 and CXCR7 were higher.

CXCR4 activation has been linked to ERK1/2, Akt, and FAK phosphorylation [9], which are important pathways regulating the survival, proliferation and invasion of tumor cells. Our data demonstrate that SDF-1α induced the phosphorylation of ERK1/2 and Akt in LN229 and LN308 glioma cells that displayed CXCR7-mediated migration towards SDF-1α. This was mediated by CXCR7, as knockdown of CXCR7 expression decreased the levels of SDF-1α-induced phosphorylation of ERK1/2 and Akt. These data have important implications, because ERK1/2 and Akt pathways are frequently upregulated in several cancers and there are ongoing efforts exploring both pathways as potential therapeutic targets. For instance, positive staining for phosphorylated ERK1/2 is observed in a large percentage of gliomas, but not in normal brain. Indeed, inhibition of MAPK signaling by the inhibitor sorafenib suppressed development of malignant glioma in an orthotopic mouse model [13].

Functionality of CXCR7 has long been the source of controversy. To date, several studies have yielded puzzling results. While some reports suggest a decoy activity, others indicate a signaling activity for CXCR7. Burns and colleagues showed that ligand activation of CXCR7 failed to induce typical chemokine responses, such as cell migration and calcium mobilization [8]. This was supported by studies in zebrafish that showed CXCR7 functions primarily by sequestering SDF-1α to shape the extracellular chemokine gradient and provide directional migration [14]. By contrast, Wang and coworkers provided evidence that CXCR7 induces invasiveness of prostate cancer cells and activates Akt [7]. Invasiveness of hepatocellular carcinoma cells is also mediated by CXCR7 [15]. There is evidence that ligand binding to CXCR7 activates MAPK through β-arrestin and thus the receptor is functional [16]. CXCR7 is implicated in survival and proliferation of breast and lung cancer cells [6]. Moreover, studies have unraveled that CXCR7 regulates interneuron migration [17], and is involved in transendothelial migration [18]. A recent study reported that CXCR7 modulates chemokine responsiveness in migrating neurons by regulating CXCR4 protein levels [19]. CXCR7 is also a functional receptor in primary rodent astrocytes and controls proliferation and migration towards SDF-1α through G_i/o_ proteins [20]. CXCR7 is involved in mediating anti-apoptotic events in glioma cells as well [21,22]. A functional interaction is evident between CXCR4 and CXCR7. In GBM cell lines, CXCR7 controls proliferation through a functional cross-talk with CXCR4 [23], and in the developing rat brain, a cross-talk between CXCR4 and CXCR7 might account for the regulation of SDF-1α-dependent neuronal development [24]. In breast cancer cells, inhibition of CXCR7 was shown to reduce the growth and metastasis of CXCR4-positive cells [25]. Targeting of CXCR7 also inhibits SDF-1α/CXCR4-mediated transendothelial migration of human tumor cells [26].

We now provide evidence that CXCR7 is induced by hypoxia, and mediates the migration of glioma cells towards SDF-1α in hypoxic conditions. Our data reveal that both CXCR4 and CXCR7 are required for migration towards SDF-1α and SDF-1α-induced phosphorylation of ERK1/2 and Akt. In LN229 and LN308 glioma cells, both inhibition of CXCR4 by AMD3100 and shRNA-mediated knockdown of CXCR7 expression diminished migration towards SDF-1α and reduced levels of SDF-1α-induced phosphorylation of ERK1/2 and Akt.

It is interesting that while both CXCR4 and CXCR7 are required for SDF-1α-induced migration of hypoxic glioma cells, blocking both CXCR4 and CXCR7 does not provide an additive effect, either with regards to migration assays or phosphorylation of ERK1/2 and Akt. Furthermore, CXCR7 can be co-immunoprecipitated with CXCR4-HA. It is probable that CXCR7 is part of a functional heterodimer, together with CXCR4, which mediates the migration of glioma cells towards SDF-1α under hypoxic conditions. Functional CXCR4/CXCR7 heterodimerization has previously been reported in HEK293T cells and glial cells [27-29].

GPCRs can exist as monomers, homodimers or heterodimers and these conformations might have important implications in downstream signaling and the design of pharmacological inhibitors. It has been demonstrated that heterodimers can activate signaling pathways that differ from those activated by homodimers [30]. Our previous data showed that CXCR4 inhibition by AMD3100 decreased the levels of SDF-1α-induced phosphorylation of FAK in LN308 glioma cells [9]. Conversely, the data that we present here show that knockdown of CXCR7 expression in LN308 glioma cells did not affect the levels of SDF-1α-induced phosphorylation of FAK. Activation of FAK following exposure to SDF-1α might therefore depend on CXCR4 alone. This scenario has obvious implications for drug discovery. Heterodimers may be considered as distinct structural and functional entities, which might influence drug affinity and efficacy. A better understanding of how heterodimers are regulated, their function, and pathophysiological significance may help us exploit them as novel drug targets for improved therapeutics.

It is of note that, as mentioned above, CXCR4 and CXCR7 are present on both tumor cells and vascular cells. This suggests that paracrine signaling mechanisms between these two cell types might be in effect. Such mechanisms could affect several aspects of tumor biology, including angiogenesis, migration, survival and proliferation.

## Conclusions

In summary, the studies described here show that CXCR7 is a hypoxia-responsive mediator of SDF-1α-induced glioma cell migration and support the development of therapeutic agents for the pharmacological inhibition of CXCR4 and CXCR7 to control glioma cell migration.

## Methods

### Cell culture and reagents

Human glioma cell lines U87MG, LN229 and LN308 were obtained from ATCC. The human embryonic kidney 293T (HEK293T) cells, used for lentivirus production studies were kindly provided by Dr. Pagano, New York University. Cell lines were cultured in 5% CO_2_ at 37°C in Dulbecco’s Modified Eagle Medium (DMEM, Cellgro). The medium was supplemented with 10% fetal bovine serum (FBS, Atlanta Biologicals), 1% penicillin and streptomycin, and 2 mM glutamine (Gibco BRL). For hypoxic exposure, cells were placed in a sealed Modular Incubator Chamber (Billups-Rothenberg Inc.) flushed with 1% O_2_, 5% CO_2_, and 94% N_2_. Recombinant human SDF-1α/CXCL12 (R&D Systems Inc.) was prepared in 0.1% BSA in PBS and stock solution (100 μg/ml) was stored at −20°C. AMD3100, a CXCR4 inhibitor [9] (Sigma-Aldrich), was prepared in PBS (5 mg/ml) and kept at 4°C until used.

### Western blot analysis

Cells were lysed in RIPA buffer supplemented with protease inhibitors [10]. Protein quantitation and electrophoresis were performed as previously described [10]. Western blot analysis was performed with the following antibodies: rabbit anti-CXCR4 polyclonal antibody 1:500 (43 kDa; Imgenex), rabbit anti-CXCR7 polyclonal antibody 1:1000 (52 kDa; Abcam), rabbit anti-HIF-1α polyclonal antibody 1:500 (120 kDa; Bethyl Laboratories, Inc.), mouse anti-p-ERK1/2 monoclonal antibody 1:1000 (44/42 kDa; Santa Cruz Biotechnology, Inc.), rabbit anti-ERK1/2 polyclonal antibody 1:1000 (44/42 kDa; Cell Signaling Technology, Inc.), rabbit anti-p-Akt polyclonal antibody 1:1000 (60 kDa; Cell Signaling Technology, Inc.), rabbit anti-Akt polyclonal antibody 1:1000 (60 kDa; Cell Signaling Technology, Inc.), rabbit anti-p-FAK polyclonal antibody 1:1000 (125 kDa; Abcam), rabbit anti-FAK polyclonal antibody 1:1000 (125 kDa; Abcam) and mouse anti-actin monoclonal antibody 1:20,000 (42 kDa; clone C4, Chemicon International, Inc.). Donkey anti-rabbit and anti-mouse IgG horseradish peroxidase-conjugated secondary antibodies (Amersham Life Pharmacia Biotech) were used at 1:2500 dilution. Immunodetection was carried out with the Supersignal West Pico Chemiluminescent Reagent (Thermo Fisher Scientific). Visualization and densitometry of protein bands were performed with the National Institutes of Health (NIH) Image software (version 1.62). In Figure 1, measurements of CXCR7 levels were normalized to loading control, and in Figures 4 and 5, measurements of p-ERK 1/2, p-AKT and p-FAK were normalized to total ERK 1/2, AKT, and FAK, respectively.

### Migration assay

BD Biocoat chambers (BD Bioscience Discovery Labware) with 8-μm pore size polycarbonate filter inserts for 24-well plates were used according to the manufacturer’s instructions and as described [10]. Briefly, shRNA-infected cells (1 × 10 [5]) were seeded onto the upper chambers in 400 μl of DMEM medium with 1% FBS in the presence or absence of 100 nM of AMD3100 and placed into wells containing 600 μl of complete medium with or without SDF-1α (100 ng/ml) to induce cell migration. The migration chambers were incubated for 8 h in normoxic or hypoxic conditions at 37°C. After incubation, the inserts were fixed and stained and the number of migrating cells was counted as described [10]. Each assay was performed in duplicate and repeated two times with similar results. The data from independent experiments were pooled for statistical analysis.

### Lentivirus production and infection of glioma cells

Five different shRNA sequences directed against CXCR7 were purchased from Open Biosystems and used to knockdown CXCR7 expression in U87MG, LN229 and LN308 glioma cells. Recombinant lentiviruses were produced by cotransfecting HEK293T cells with the lentivirus expression vector (pLKO.1 puro) and packaging plasmids (Δ8.9 and vsv-g) using Fugene 6 (Roche Diagnostics) as a transfection reagent. Infectious lentiviruses were collected at 24, 48 and 72 h after transfection and the pooled supernatants centrifuged to remove cell debris and filtered through a 0.45 μm filtration unit. Glioma cells were infected and stable transfectants were selected in puromycin for 7 days. After this time, cells were expanded and exposed to normoxic or hypoxic conditions to test for CXCR7 downregulation. Two of the five shRNA sequences (S4 and S5) efficiently downregulated CXCR7 expression in glioma cells based on Western blot analysis and were used for further investigations.

### Immunoprecipitation

For immunoprecipitation, 60%-80% confluent LN229 and LN308 glioma cells were transfected with 5 ug of HA-tagged CXCR4 (kindly provided by Dr. Marchese, Loyola University Chicago) or empty vector as control using X-tremeGENE HP DNA Transfection Reagent (Roche) according to the manufacturer’s protocol. After 24 h, cells were lysed in ice-cold NP-40 buffer [50 mM Tris- HCl pH 7.5 containing 0.5% Igepal CA-630, 150 mM NaCl, 10% glycerol, 1 mM EDTA, 5 mM MgCl_2_ and protease inhibitor cocktail (Sigma)]. After preclearing, lysates were incubated with anti-HA antibodies (Covance) at 4°C for 1 hour, followed by another 1 hour incubation period in the additional presence of protein G Sepharose beads (4B, Invitrogen). The beads were washed three times in lysis buffer and then resuspended in sample buffer. Samples were later subjected to Western blot analysis using anti-HA and anti-CXCR7 (Abcam) antibodies.

### Statistical methodologies

Statistical significance was determined by unpaired t-test (GraphPad Prism Software).

## Competing interests

The authors declared that they have no competing interest.

## Authors’ contributions

ME designed and did the experiments and drafted the manuscript. DZ conceived the study and critically revised the manuscript. YS assisted ME and DZ with the response letter. All authors read and approved the final version of the manuscript.

## Pre-publication history

The pre-publication history for this paper can be accessed here:

http://www.biomedcentral.com/1471-2407/13/347/prepub
